# Intravenous vitamin C in adults with sepsis in the intensive care unit: still LOV’IT?

**DOI:** 10.1186/s13054-022-04106-w

**Published:** 2022-07-30

**Authors:** Christian Stoppe, Jean-Charles Preiser, Daniel de Backer, Gunnar Elke

**Affiliations:** 1 Department of Anaesthesiology, Intensive Care, Emergency and Pain Medicine, University Hospital Wuerzburg, Josef-Schneider-Straße 2, 97080 Wuerzburg, Germany; 2 Medical Direction, Erasme University Hospital, Universtié Libre de Bruxelles, Brussels, Belgium; 3 Department of Intensive Care, CHIREC Hospitals, Université Libre de Bruxelles, Brussels, Belgium; 4 Department of Anesthesiology and Intensive Care Medicine, University Medical Center Schleswig-Holstein, Campus Kiel, Arnold-Heller-Str. 3 Haus 12, 24105 Kiel, Germany

The recent results of the lessening organ dysfunction with vitamin C (LOVIT) randomized controlled trial (RCT) have challenged the potentially beneficial role and brought concerns on the safety of high-dose vitamin C in patients with sepsis. Septic patients admitted to the intensive care unit (ICU) received either high-dose vitamin C or placebo. As opposed to placebo, vitamin C was associated with an increased occurrence of the primary endpoint (persistent organ dysfunction plus death) [1]. While these results may suggest the end of the vitamin C story, several aspects suggest that LOVIT is just one piece of the puzzle and that the baby should not be thrown out with the bathwater.

Regarding LOVIT, it should be noted that although the primary endpoint was met, there were no differences in its individual components. Imbalances in baseline characteristics may have contributed to the observed differences. Patients in the intervention group had 10% higher lactate levels, were more often in shock and mechanically ventilated already at baseline. Thus, compared to placebo, patients receiving vitamin C appeared to be sicker, overall contributing to the higher risk of organ dysfunction.

The results of LOVIT differ from previous RCTs, where beneficial effects of vitamin C were observed: Vitamin C may restore vascular responsiveness to vasoactive agents, improve microcirculatory blood flow, preserve endothelial function, augment bacterial defense and prevent apoptosis.^2^ Due to its redox-potential and powerful antioxidant capacities, vitamin C may modify the inflammatory cascade and related organ dysfunctions [2]. Observational studies demonstrated low vitamin C levels to be associated with organ dysfunctions in septic patients [3, 4]. As humans are incapable to synthesize and store vitamin C, supplementation to replete vitamin C pool is imperative [5].

The clinical significance of high-dose vitamin C given as a "sepsis cocktail” with hydrocortisone and thiamine was popularized by Marik et al. [6]. This cocktail significantly reduced hospital mortality, time on vasopressor and organ injury. Consequently, numerous RCTs assessing the effects of IV vitamin C have been performed in critically ill patients followed by several systematic reviews and meta-analyses (SRMA) [7]. Some demonstrated benefits including lower mortality, less organ dysfunction and reduced duration on vasopressor support in those patients receiving high-dose vitamin C [7]. None of these trials showed evidence of harm of high-dose vitamin C in septic or non-septic critically ill patients [8], with exception of one study using a prolonged continuous infusion of vitamin C that indicated an increase in acute kidney injury (AKI) [9]. No evidence of increased AKI was observed in LOVIT.

The most recent updated SRMA demonstrated beneficial effects on 30-day and hospital mortality in 4366 patients, while a detrimental effect was observed at 90 days in an analysis including only a subset of 1722 patients and for which LOVIT contributed to 58% [10].

The LOVIT investigators could not provide an explanation for their findings. We offer further thoughts, which should carefully be considered for the interpretation of the received findings and planning of future studies (Table 1).

**Table 1:** Research questions regarding vitamin C supplementation in critically ill (septic) patients

Problem	Comments
Is the dose of vitamin C adequate?	Correction of severe vitamin C deficiencies is essential SRMA suggest that higher doses may be beneficial
Should vitamin C be triggered by vitamin C levels?	Measurements of vitamin C are cumbersome, take time and are not broadly available The target level (normalization vs supratherapeutic levels) is not yet defined Benefits of vitamin C may not be restricted to patients with vitamin C deficiency (i.e., endothelial function is improved independently of vitamin C levels)
What is the optimal timing for vitamin C initiation?	The timing of supplementation is of paramount importance and may often have been too late to translate in clinically meaningful effects Vitamin C should probably be given timely after onset of critical illness (e.g., 24 hours), whereas more research is needed
What is the optimal treatment duration of vitamin C?	The 4 days period has been selected arbitrarily
Should vitamin C be supplemented with or without co-supplementation of thiamine and hydrocortisone?	Current SRMAs do not suggest a beneficial effect of thiamine and hydrocortisone co-administration
Which biomarkers should be used to monitor vitamin C effectiveness?	Appropriate surrogate markers for vitamin C, which reflect the biological response to vitamin C are still missing The oxidation–reduction potential (ORP) has early been reported to adequately reflect the patients` oxidative response
Which markers should be used to monitor vitamin C potential adverse effects	Markers of AKI should be monitored Patients with urinary oxalate crystals or Glucose-6-phosphate dehydrogenase (G-6-PD) deficiency should not receive high-dose vitamin C
Which critically ill (patients may benefit from vitamin C administration?	Heterogeneity in vitamin C response between RCT suggest that patient population may be important. None of the yet reported baseline conditions help to identify the ideal target subgroup
Which clinical relevant outcome measure adequately captures the effects of Vitamin C?	Minimal data available. Early changes in SOFA score seems not sensitive enough Outcome measures beyond the ICU stay such as functional recovery of critically ill patients should receive more attention

First, assuming a time dependent production and release of reactive oxygen species (ROS), the benefit of vitamin C may depend on the redox- and inflammation balance in the early acute inflammatory phase of sepsis [11]. Besides hyperinflammation with overwhelming release of ROS, patients with sepsis including SARS-CoV-2 commonly show a period of relative immunosuppression [12]. In the LOVIT trial, patients were excluded if > 24 h in the ICU; however, 13.3% of patients in the IVVC group already spent 49 h in another hospital’s ICU, so that the acute onset of sepsis may have occurred earlier. Thus, the initiation of the treatment may have been started too late being mainly delivered after the initial cytokine storm, and probably given here at relatively too high dose, potentially negating any biologically plausible antioxidant treatment benefits.

Second, in most of the trials, patients were included largely based on undifferentiated phenotypes, likely having a different mortality risk and also different treatment response [12, 13]. Thus, imbalances in sepsis phenotypes may have contributed to the heterogeneity in response to vitamin C in the different trials. Third, no surrogate markers of vitamin C were measured and the average vitamin C level (measured in a sub-cohort) was in the normal range, whereas patients with vitamin C deficiency are known to most likely to benefit from a supplementation. The absence of severe deficiencies, or biological surrogate markers that identify patients, which benefit from the intervention may provide explanations, why multiple RCTs have repeatedly failed to demonstrate clinical benefits.^13^ It has been hypothesized that not all patients show the same response to an intervention, so that the future concept of a personalized therapy should be adapted to interindividual “metabotypes” based on patient’s illness severity, level of inflammation and oxidative stress capacity (“sweet spot”), respectively [14].

So how do we get there? It is under current debate that we have to move beyond syndromic characterization of the underlying disease of critical illness and to develop disease models based on shared pathophysiological patterns [15]. While position papers and consensus conferences try to provide better guidance during times were several RCTs having failed to demonstrate beneficial effects, a combination of theoretical and practical considerations across key domains such as the patients' individual insult and biological response deserves more attention. Ongoing larger RCTs in different cohorts including COVID-19 (LOVIT-COVID, REMAP-CAP), burn (VICTORY), post-cardiac arrest (VITaCCA) and/or cardiac surgery patients (advanceCSX) may help to answer the question whether vitamin C treatment is effective in more specific patient cohorts.

Vitamin C—lov’it or not anymore? Most recent evidence does not support the routine administration of high-dose vitamin C in septic patients. Heterogeneity in outcome between various studies suggests that certain subsets of patients may benefit from vitamin C. Further trials will focus on a more personalized approaches to identify which critically ill patients respond positively to a certain intervention. These should also better define the optimal dose and duration of therapy, better assessment of the risk-to-benefit ratio, evaluate the underlying mechanism and consider which clinical outcomes are likely to be improved by the intervention.

## Data Availability

Not applicable.

## Abbreviations

ICU: Intensive care unit
LOVIT: Lessening organ dysfunction with vitamin C
RCT: Randomized controlled trial (RCTs)
RNS: Reactive nitrogen species
ROS: Reactive oxygen species
SRMA: Systematic review and meta-analysis
